# *Nosema ceranae* causes cellular immunosuppression and interacts with thiamethoxam to increase mortality in the stingless bee *Melipona colimana*

**DOI:** 10.1038/s41598-020-74209-3

**Published:** 2020-10-12

**Authors:** José O. Macías-Macías, José C. Tapia-Rivera, Alvaro De la Mora, José M. Tapia-González, Francisca Contreras-Escareño, Tatiana Petukhova, Nuria Morfin, Ernesto Guzman-Novoa

**Affiliations:** 1 Centro de Investigaciones en Abejas (CIABE), Centro Universitario del Sur, Universidad de Guadalajara, Enrique Arreola Silva 883, Cd., Guzman, Jal. Mexico; 2 School of Environmental Sciences, University of Guelph, 50 Stone Road E., Guelph, ON N1G2W1 Canada; 3 Depto. de Producción Agrícola, Centro de Investigaciones en Abejas (CIABE), Centro Universitario de la Costa Sur, Universidad de Guadalajara, Independencia Nal. 161, Autlan, Jal. Mexico; 4 Dept. Population Medicine, University of Guelph, 50 Stone Road E., Guelph, ON N1G2W1 Canada

**Keywords:** Ecology, Microbiology

## Abstract

The microsporidian parasite *Nosema ceranae* and neonicotinoid insecticides affect the health of honey bees (*Apis mellifera*). However, there is limited information about the effect of these stressors on other pollinators such as stingless bees (Hymenoptera: Meliponini). We examined the separate and combined effects of *N. ceranae* and the neonicotinoid thiamethoxam at field-exposure levels on the survivorship and cellular immunity (hemocyte concentration) of the stingless bee *Melipona colimana*. Newly-emerged bees were subjected to four treatments provided in sucrose syrup: *N. ceranae* spores, thiamethoxam, thiamethoxam and *N. ceranae*, and control (bees receiving only syrup). *N. ceranae* developed infections of > 467,000 spores/bee in the group treated with spores only. However, in the bees subjected to both stressors, infections were < 143,000 spores/bee, likely due to an inhibitory effect of thiamethoxam on the microsporidium. *N. ceranae* infections did not affect bee survivorship, but thiamethoxam plus *N. ceranae* significantly increased mortality. Hemocyte counts were significantly lower in *N. ceranae* infected-bees than in the other treatments. These results suggest that *N. ceranae* may infect, proliferate and cause cellular immunosuppression in stingless bees, that exposure to sublethal thiamethoxam concentrations is toxic to *M. colimana* when infected with *N. ceranae*, and that thiamethoxam restrains *N. ceranae* proliferation. These findings have implications on pollinators’ conservation.

## Introduction

Populations of managed and unmanaged bees, which are essential pollinators of crops and wild plant species, have been declining at unprecedented rates in recent decades. Honey bee (*Apis mellifera*) colonies, for example, have been collapsing at a rate of over 30% annually in North America and some European countries during the last 13 years, due to multiple factors^1,2^. It is concerning that at the same time that bee populations have been declining the demand for bee pollination of crops has increased^3^, which may lead to a pollination crisis. Pathogens and pesticides have been identified as frequent culprits of bee mortality^2,3^. Among pathogens, the fungus *Nosema ceranae* has been associated with honey bee mortality and colony depopulation^4–7^, while numerous studies have identified pesticides, particularly neonicotinoid insecticides, as important drivers of bee declines^3^.

*Nosema ceranae* is a microsporidian obligate parasite that infects the midgut’s epithelial cells of the honey bee, impairing digestive functions and the absorption of nutrients^8^. *N. ceranae* infections may reduce the lifespan and colony populations of honey bees^9^ and may also affect their physiology and behavior^10^. For example, infected bees become precocious foragers and their homing and foraging ability are reduced compared with non-infected bees^11–15^, which potentially compromises colony fitness by reducing the collection of food resources. *N. ceranae* may also suppress humoral immune responses in honey bees^16–20^ or may trigger them in certain cases^20–22^. However, not much is known about how the parasite affects cellular immunity carried out by hemocytes, particularly in non-*Apis* bees.

*Nosema ceranae* has also been detected in bee species other than honey bees, including bumble bees^23–27^ in different continents, and stingless bees in Australia^28^ and South America^29^. It is believed that the pathogen is transmitted from honey bees to wild bees via flowers^28,30^, but little is known about whether *N. ceranae* multiplies and causes damage to wild bees or if these are only accidental reservoirs and vectors of the microsporidium. The presence of this pathogen in wild bees may be threatening their health and potentially that of other wild pollinators. For example, in bumble bees, Graystock et al.^25^ inoculated workers of *Bombus terrestris* with *N. ceranae* spores and were able to show that nearly 100% of the bees became infected and lived shorter lives compared to non-inoculated bees.

Another factor frequently implicated in recent cases of bee mortality are the neonicotinoid insecticides^2,3,31^, which interfere with nicotinic acetyl-choline receptors and thus can affect multiple neural processes in target and non-target insects, such as many pollinators. It has been found that these pesticides may impair foraging behavior in honey bees and bumble bees, resulting in reduced pollen collection^32,33^, and can also affect their humoral immunity^34–36^. Synergic effects of neonicotinoid insecticides and *N. ceranae* associated to increased honey bee mortality have been demonstrated^20,37–41^, but not much is known about synergistic effects of insecticides and *N. ceranae* on the cellular immunity of bees, other than honey bees.

Clearly, honey bees are negatively impacted by *N. ceranae* and neonicotinoid insecticides alone or in combination, but we know very little about how they affect other bee species, particularly in the tropics. Few studies have shown that stingless bees, social bees lacking stingers, are negatively impacted by neonicotinoid insecticides^42,43^ and can be vectors of *N. ceranae* spores^28,29^, but we do not know if *N. ceranae* is capable of infecting and proliferating in them or if they could be adversely affected if exposed to both stressors, something likely to occur in natural and managed ecosystems. This is important, not only because stingless bees play a significant role as pollinators of flowering plants in the tropics, but also because several species of stingless bees have been managed for honey and cerumen production since ancient times, constituting a source of income for indigenous populations in the Americas^44,45^. Thus, it is critical that they maintain strong and healthy populations.

*Melipona colimana* Ayala 1999 is a species of stingless bee endemic to oak and pine tree forests in the occidental mountains of Mexico^46^. There is little information about the health status of this bee species, but it is known that honey bee viruses can infect them and replicate in them^47^. However, we do not know if other pathogens, like *N. ceranae*, can infect and multiply in these bees, or if they might simply be occasional vectors of the microsporidium. We do not know either if *M. colimana* bees are affected by neonicotinoid insecticides. In fact, there are less than 10 studies about *M. colimana*, but one study has shown evidence suggesting that populations of this stingless bee are declining^48^. The specific culprits of this decline are unknown.

This study was conducted to determine if *N. ceranae* can infect and proliferate in *M. colimana* bees, as well as to examine the effect of a neonicotinoid insecticide, thiamethoxam, alone or in combination with *N. ceranae* on their survivorship and cellular immunity.

## Results

### Food consumption

Food consumption did not differ between treatments (*H* = 1.64, *df* = 3, *p* = 0.65). Bees of the different groups consumed 22.0 ± 2.2, 23.0 ± 2.4, 18 ± 2.8, and 20.6 ± 1.4 mg of sucrose syrup/day/bee for the control, *N. ceranae*, thiamethoxam and *N. ceranae* + thiamethoxam treatments, respectively.

### Infectivity and intensity of *N. ceranae* infections

**Figure 1:**
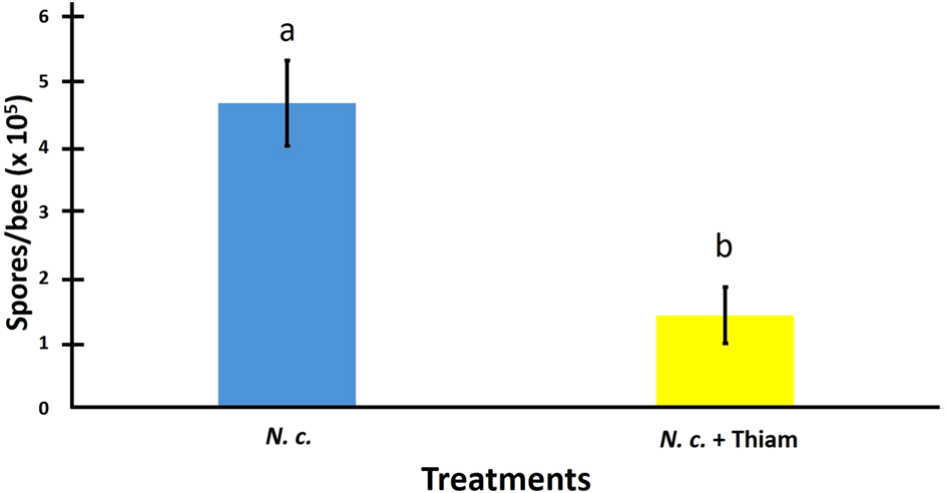
Mean number of *Nosema ceranae* (*N.c*.) spores per individual (± SE) for *Melipona colimana* bees at 14 days post-treatment with an inoculum of 50,000 spores/bee or additionally exposed to a sublethal concentration of thiamethoxam in sucrose syrup (4.2 × 10^–3^ ng/µl; *N.c*. + Thiam). Significant differences between the two treatments based on a two-sample Wilcoxon test are shown with different literals above bars.

A higher percentage of bees became infected with *N. ceranae* spores when inoculated with the fungus only (66.2 ± 16.9) than when bees also received a thiamethoxam treatment (51.6 ± 18.8), but this difference was not significant (*W* = 23.5, *p* = 0.412). However, the spore counts for bees treated with *N. ceranae* only, were over nine times higher than the initial individual inoculum, and three times and significantly higher than the spore counts for bees treated with *N. ceranae* + thiamethoxam (*W* = 507, *p* = 0.001; Fig. 1).

### Survivorship

**Figure 2:**
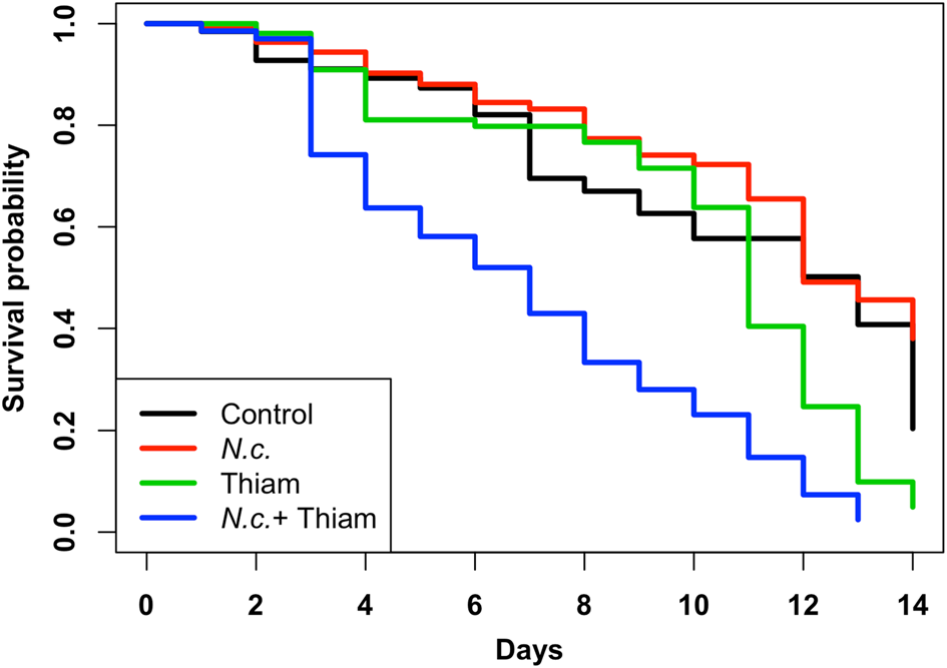
Survivorship probability of *Melipona colimana* bees not treated (Control in black), or in response to *Nosema ceranae* infection (*N.c*. in red), exposure to a sublethal concentration of thiamethoxam (4.2 × 10^–3^ ng/µl) in sucrose syrup (Thiam  in green) and the combination of both factors (*N. c*. + Thiam in blue) during 14 days. Survival functions were estimated with the Kaplan–Meier method.

Bees treated with *N. ceranae* only, thiamethoxam only and control bees did not differ in overall survivorship (*p* > 0.05), but bees treated with *N. ceranae* + thiamethoxam had a significantly lower probability of surviving and a shorter lifespan than bees of the other treatments (*p* < 0.01). Additionally, at the end of the experiments, the proportions of live bees of the control group and that of bees treated with *N. ceranae* only, were significantly higher than the proportion of live bees treated with thiamethoxam only, or the combination of thiamethoxam + *N. ceranae* (*p* < 0.01; Fig. 2).

### Hemocyte concentrations

**Figure 3:**
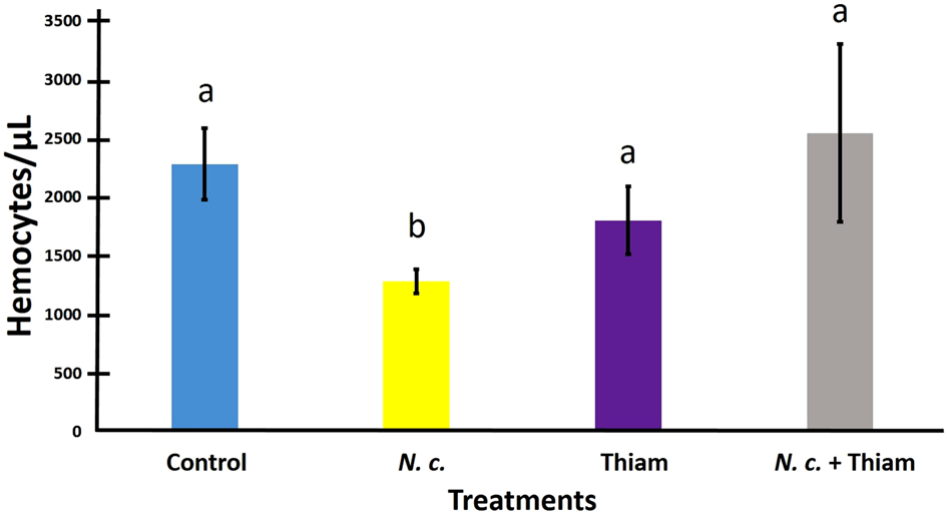
Mean hemocyte concentration/µL of hemolymph (± SE) in *Melipona colimana* bees not treated (Control), or in response to *Nosema ceranae* infection (*N.c*.), exposure to a sublethal concentration of thiamethoxam (4.2 × 10^–3^ ng/µl) in sucrose syrup (Thiam) and the combination of both factors (*N. c*. + Thiam) during 14 days. Different letters indicate significant differences based on ANOVA and Dunnett tests of logarithm transformed data. Actual, untransformed values are depicted.

Bees treated with *N. ceranae* only had significantly lower concentrations of hemocytes in the hemolymph than bees of the rest of the treatments, among which there were no differences (*F*_3, 71_ = 5.42, *p* = 0.002; Fig. 3).

## Discussion

*Melipona colimana* bees were negatively affected by the stressors tested alone and in combination. However, there were no differences for food consumption between treatments. In other bee species such as *A. mellifera* and *B. terrestris*, neonicotinoids seem to stimulate sucrose syrup consumption^49,50^.

Spores of *N. ceranae* were detected in more than 50% of the inoculated bees at 14 dpt and the number of spores in bees treated with *N. ceranae* only were at least nine times higher than the initial individual inoculum, indicating that *N. ceranae* was able to replicate in *M. colimana* bees. To the best of our knowledge, this is the first report of *Nosema* spp. replication in stingless bees, at least in the Americas. A recent study screened for pathogens in three stingless bee species in Brazil, finding *N. ceranae* in the workers, but were not able to detect it in their midguts, which indicates that those species of stingless bees were potentially vectors of the microsporidium^51^. In our study, spores were extracted from the midgut of the bees and proliferation of spores suggests that *M. colimana* bees maybe affected by *N. ceranae*. Additionally, the infection intensity of bees treated with *N. ceranae* spores only, was significantly different and at least three times higher than that of bees treated with both stressors, which suggests that thiamethoxam may suppress or restrain the proliferation of *N. ceranae* in *M. colimana* bees. Again, this is the first evidence of a potential inhibitory effect of a neonicotinoid on *N. ceranae* proliferation in stingless bees. However, a similar result had been previously reported from studies in which thiacloprid and imidacloprid had a negative effect on *N. ceranae* proliferation in *A. mellifera*^37,39^. This report in *M. colimana* and those in *A. mellifera*, reinforce the argument of an inhibitory effect of neonicotinoid pesticides on *N. ceranae* replication. It may be that thiamethoxam and other neonicotinoids have anti-fungal activity, which has been shown in studies with other organisms where insecticides decreased the germination or proliferation of fungi^52,53^. However, it is important to consider that the effects of neonicotinoid insecticides on *N. ceranae*, an obligate intracellular parasite, may be different than in more primitive fungi. The insecticides might also promote the activation of gut defenses by changing the microbiota composition of infected bees, or by having a protective effect on midgut epithelial cells as evidenced by immunohistochemical analysis for thiamethoxam^41^. An alternative explanation of these results is that the bees subjected to both stressors lived less long and thus the *N. ceranae* infection in these bees could not increase to the levels found in bees fed just *N. ceranae* spores. Other studies have shown antagonistic results depending on the pesticide used. For example, Vidau et al.^38^ reported a reduction of *N. ceranae* spores in bees exposed to fipronil, which agrees with our results, but also found an increase of spores when bees were exposed to thiacloprid. However, they did not explain the potential basis for this antagonistic result. Contrary to the results of the above studies, Pettis et al.^54^ found that worker honey bees from neonicotinoid-exposed colonies developed more intense *N. ceranae* infections than bees from non-exposed colonies. The inconsistency of these results may be due to differences in experimental setups (colony vs. cage experiments), differences related to the specific pesticide used, differences in susceptibility of the experimental bees, or differences in *N. ceranae* strains. These and other potential hypotheses that could help explain the above results, warrant further investigation.

*Nosema ceranae* did not affect survivorship in *M. colimana* bees up to 14 dpt. However, thiamethoxam at a sublethal concentration reduced the survival of the bees by 12 dpt. The lowest survivability was for the bees treated with both stressors, which suggests a detrimental synergistic effect due to the interaction of *N. ceranae* and thiamethoxam on the lifespan of *M. colimana*. Conversely to our findings, it has been reported that *N. ceranae* may shorten the lifespan of *A. mellifera* and *B. terrestris*^5,25^. The reason why the fungus did not decrease the survivorship rate of *M. colimana* bees could be because the parasite’s infection intensity was lower compared with levels found in *A. mellifera*^55,56^, indicating a possible lower susceptibility of *M. colimana* to *N. ceranae* relative to honey bees, or perhaps to a loss in spore viability because the bees were incubated at a lower temperature (24 °C) than that of *A. mellifera* brood nests. However, infection intensity was sufficiently high to impair cellular immune responses (see below). Clearly, more studies are needed to shed light on the basis for the susceptibility of different bee species, including *M. colimana*, to *N. ceranae*.

Regarding the toxic effect of thiamethoxam at sublethal concentrations, the results of this study agree with previous reports showing reduced lifespans of other stingless bee species^42,43^. Thus, this study supports the notion that neonicotinoid insecticides are more detrimental to stingless bees at sublethal concentrations than *N. ceranae* infections. Furthermore, the combination of *N. ceranae* and thiamethoxam resulted in the lowest survivorship rates of *M. colimana* bees, suggesting a detrimental interaction of both stressors on the length of life of the stingless bees. Interactions between *N. ceranae* and neonicotinoid insecticides have resulted in high mortality rates of honey bees in some studies^20,37,39,40^, which agrees with our findings, but Retschnig et al.^57^ did not find such interactions in a study conducted at the colony level. Instead, they reported detrimental effects of the pesticide and parasite alone. These inconsistent results of different studies highlight the need for more research on the topic in different bee species using colonies, and not only cages in laboratory experiments.

Apparently, *N. ceranae* caused cellular immunosuppression in *M. colimana* bees that were inoculated with spores only but not in bees that were treated with both stressors. This is the first report that provides evidence suggesting that *N. ceranae* may not only infect stingless bees but also reduce their immune defenses. In honey bees, Alaux et al.^37^ did not find significant effects of *N. ceranae* infections on hemocyte counts after 10 days of inoculation. Conversely, in insects of the order Orthoptera, there was a significant increase in the number of circulating hemocytes at the beginning of an infection by microsporidia, which tended to sharply decrease over time^58^. In this study, hemocyte counts were determined 2 weeks after inoculation. It is possible that at the initial stages of infection, there was an increase in hemocyte concentration in the hemolymph, followed by a decrease by 14 dpt. Therefore, the low concentration of hemocytes found in *M. colimana* bees infected with *N. ceranae* could have been related to the chronic nature of the exposure. Cellular defenses respond before humoral immune defenses to fungal infections, with hemocytes encapsulating pathogens or forming nodules around tissue infected by microsporidia in Lepidoptera insects^59^. If similarly, hemocytes of *M. colimana* bees encapsulate tissues infected by *N. ceranae* during the early stages of infection, it subsequently would lead to a decrease in the number of circulating hemocytes in later stages of the infection. This scenario agrees with findings by Antúnez et al.^16^, who found an increase in PPO expression in *Nosema* infected bees, which is related to encapsulation and melanization by hemocytes. Additionally, Gilliam and Shimanuki^60^ noticed that the hemocytes that engulfed *N. apis* spores, died, which could also partially explain a decrease in the number of circulating hemocytes after several days of the infection becoming established. Regardless of the above potential explanations, the reason why *N. ceranae* may have suppressed cellular immunity in *M. colimana* is beyond the objectives of this study and thus, remains to be investigated. Further studies should analyze cellular immune responses at different time points during the process of *N. ceranae* infection in the bees.

Thiamethoxam alone did not affect cellular immunity in *M. colimana* bees at the concentration used in this study. Similarly, another study found that sublethal exposure to imidacloprid does not alter cellular immunity in the stingless bee *Melipona quadrifasciata*^61^. In other bee species like *A. mellifera*, exposure to neonicotinoid insecticides has resulted in a reduction of hemocytes in exposed bees^62^. It is difficult to explain why neonicotinoid pesticides may cause cellular immunocompetence in honey bees and not in stingless bees, but these opposite results could be a consequence of differences in levels of exposure to the insecticides, as well as to differences in species susceptibility to the effect of the pesticides. Future studies should test different doses of various pesticides in multiple bee species to better understand the nature of these differences in neonicotinoid impact on cellular immune responses. Likewise, the combination of the two stressors did not result in reduced cellular immune responses in *M. colimana* bees, which could be due to the relatively low infection intensity of *N. ceranae* in the bees from this treatment. This was likely a consequence of an inhibitory effect of the insecticide on the parasite’s proliferation, as previously discussed.

In conclusion, this is the first study providing evidence that *N. ceranae* may infect and replicate in stingless bees in the Americas and that it may inhibit cellular immunity. Thiamethoxam seems to restrain the replication of *N. ceranae* but may be toxic to *M. colimana* bees at sublethal concentrations, particularly in combination with *N. ceranae* infections, which could have negative implications on their populations and pollination services. Further studies should focus on determining the relative impact and synergistic effects of pathogens, neonicotinoids and other drivers of bee declines in populations of pollinators other than honey bees and bumble bees, particularly in the tropics.

## Methods

### Study site

The experiments were conducted at the Bee Research Centre of the University of Guadalajara’s Southern University Centre in Zapotlan, Jalisco, Mexico (19° 43′ 31″ N, 103° 27′ 41″ W) in a laboratory setting. Controlled conditions were needed to accurately assess the age and mortality rate of the treated bees, which is difficult to achieve under field conditions. Previous attempts to introduce marked bees in colonies of *M. colimana* have failed. Nest bees reject the marked bees (Macías-Macías, J.O., Pers. Comm.).

### Extraction and dilutions of *N. ceranae* spores

Adult honey bees were obtained from the entrance of a dozen colonies using a bee vacuum^63^ and the guts of the bees were processed to determine the presence and levels of *Nosema* spp. spores^64^. Three colonies that were highly infected (> 15,000,000 spores/bee) were used as a source of spores. Bees from those colonies were collected, pooled and frozen (− 20 °C) for < 24 h, when the spores were extracted from them. This freezing time does not affect *N. ceranae* spore viability (McGowan et al., unpublished data). *Nosema* spores were extracted as per McGowan et al.^65^ Briefly, the ventriculi of 30 bees were macerated in ddH_2_O and the macerate filtered and centrifuged at 800 × three times, with the resulting pellet suspended in ddH_2_O. After extraction, 10 µl of the spore suspension were placed in a hemocytometer to count spores^64^ to determine their concentration in the suspension. Spores were diluted to obtain a final concentration of 1 × 10^4^ spores/µl of sugar syrup to inoculate bees the same day. Subsamples of spores were analyzed for species identification^66^; all of them were *N. ceranae*.

### Thiamethoxam dilutions

To prepare the experimental dilutions of the insecticide, 10 mg of thiamethoxam (Sigma Aldrich, Toluca, Mex.) were diluted in 100 ml of dH_2_O, which was then used to prepare a subsequent dilution of 4.2 × 10^–3^ ng of thiamethoxam/µl in 50% sucrose syrup.

### Treatments

Combs containing emerging brood were obtained from three *M. colimana* colonies and placed in a polyurethane container (20 × 20 × 7 cm) inside an incubator (Luzeren, Model DHP, Prolab, Tlajomulco, Mex.) at 24 °C and 70% RH, optimal conditions of incubation for *M. colimana*^67^. The next morning, the newly emerged bees were transferred to a plastic container where they were starved for 2 h before treatment. *Nosema* spore diagnosis was performed as before in a subsample of 20, randomly chosen bees, to ensure that the bees were *Nosema* spore free. Groups of 20 newly emerged bees were randomly assigned to each of four treatments as follows: (1) control bees that were individually fed with 5 µl of 50% sucrose syrup using a micropipette (Eppendorf, Mississauga, ON, CA), (2) bees fed 5 µl of sucrose syrup containing 10,000 N*. ceranae* spores/µl, (3) bees fed 5 µl of sucrose syrup that were later allowed to consume sucrose syrup containing thiamethoxam at a concentration of 4.2 × 10^–3^ ng/µl, (4) bees fed 5 µl of sucrose syrup containing *N. ceranae* spores (10,000 N*. ceranae* spores/µl) and that were later allowed to consume sucrose syrup with thiamethoxam as above. Each group of bees belonging to a treatment was introduced into a sterilized wooden hoarding cage (9.0 × 15.0 × 8.0 cm). Two plastic gravity feeders, one containing dH_2_O and the other 50% sucrose syrup either alone (for groups 1 and 2) or containing the desired dose of thiamethoxam (for groups 3 and 4), were placed on the upper part of the cages for the bees to feed ad libitum. The concentration of thiamethoxam used in this study was based on calculations made by Morfin et al.^36,68^, which would result in a field-realistic dose of the pesticide as contained in the nectar of canola grown from neonicotinoid-treated seeds. The amount of thiamethoxam that an individual bee consumed per day in sucrose syrup (18–23 µl) at the concentration used (0.0042 ng/µl) is approximately 44–57-fold lower than the estimated LD^50^ of the pesticide for honey bees^69^. The cages were maintained in an incubator at 24 °C and 70% RH during 14 days^67^. Three biological replications of the experiment were conducted.

### Health parameters

To calculate the amount of syrup consumed, the feeders with the syrup were weighed before placing them on the cages and then every day using a scientific balance (Denver Instruments Model S-403, Bohemia, NY, USA); consumption data were adjusted for daily mortality. Bee mortality was recorded daily, whereas *N. ceranae* infection, as well as hemocyte concentration in the hemolymph were quantified in each of the surviving bees at 14 days-post-treatment (dpt), except for the bees belonging to the *N. ceranae* and thiamethoxam treatment, which was done at 13 dpt, due to the low number of bees remaining alive. *N. ceranae* infections were determined as mentioned before, whereas to quantify hemocytes, each bee was pierced between the second and third dorsal tergite with an entomological pin (Bioquip, Domingues, CA, USA) to obtain with the aid of a micropipette, at least 4 µl of hemolymph. The hemolymph was spread over a microscope slide and stained with Hema 3 (Fisher Health Care Protocol, Mississauga, ON, Canada). The concentration of hemocytes in the hemolymph of the bees was determined as per Koleoglu et al.^70^. Three biological replications of the experiment were conducted.

### Statistical analyses

Data on food consumption did not satisfy the normality and homogeneity assumptions for ANOVA and could not be normalized. Therefore, they were analyzed with a Kruskal–Wallis test. Data on infectivity (proportion of bees infected with *N. ceranae*) and on *N. ceranae* infection intensity were analyzed with a non-parametric two-sample Wilcoxon test as the data were not normally distributed. Data on hemocyte counts were transformed with a natural logarithm function to satisfy the normality assumption and were then analyzed with one-way ANOVA. When differences were detected, means were separated with a Dunnett’s test. Data on survivorship were analyzed with the Kaplan–Meier method, comparing estimated survival functions. Survival curves were compared with a long-rank test. All statistical analyses were performed with R (R Development Core Team, Auckland, NZ) with a significance level set at *p* < 0.05.

## Data Availability

The data generated and related to this study are available from the corresponding author on reasonable request.

## References

[1] vanEngelsdorp D (2009). Colony collapse disorder: A Descriptive study. PLoS ONE.

[2] Guzman-Novoa E, Cork S, Hall DC, Liljebjelke K (2016). Colony collapse disorder and other threats to honey bees. One Health Case Studies: Addressing Complex Problems in a Changing World.

[3] Goulson D, Nicholls E, Botias C, Rotheray EL (2015). Bee declines driven by combined stress from parasites, pesticides, and lack of flowers. Science.

[4] Higes M, Martín R, Meana A (2006). *Nosema ceranae*, a new microsporidian parasite in honeybees in Europe. J. Invertebr. Pathol..

[5] Higes M (2008). How natural infection by *Nosema ceranae* causes honeybee colony collapse: Natural *Nosema ceranae* infection. Environ. Microbiol..

[6] Cox-Foster DL (2007). A metagenomic survey of microbes in honey bee colony collapse disorder. Science.

[7] Martín-Hernández R (2007). Outcome of colonization of *Apis mellifera* by *Nosema ceranae*. Appl. Environ. Microbiol..

[8] Goblirsch M (2018). *Nosema ceranae* disease of the honey bee (*Apis mellifera*). Apidologie.

[9] Botías C, Martín-Hernández R, Barrios L, Meana A, Higes M (2013). *Nosema* spp. infection and its negative effects on honey bees (*Apis mellifera iberiensis*) at the colony level. Vet. Res..

[10] Paris L, El Alaoui H, Delbac F, Diogon M (2018). Effects of the gut parasite *Nosema ceranae* on honey bee physiology and behavior. Curr. Opin. Insect Sci..

[11] Schneider CW, Tautz J, Grünewald B, Fuchs S (2012). RFID Tracking of sublethal effects of two neonicotinoid insecticides on the foraging behavior of *Apis mellifera*. PLoS ONE.

[12] Goblirsch M, Huang ZY, Spivak M (2013). Physiological and behavioral changes in honey bees (*Apis mellifera*) induced by *Nosema ceranae* infection. PLoS ONE.

[13] Wolf S (2014). So near and yet so far: Harmonic radar reveals reduced homing ability of *Nosema* infected honey bees. PLoS ONE.

[14] Natsopoulou ME, McMahon DP, Paxton RJ (2016). Parasites modulate within-colony activity and accelerate the temporal polyethism schedule of a social insect, the honey bee. Behav. Ecol. Sociobiol..

[15] Fleites-Ayil FA, Quezada-Euán JJG, Medina-Medina LA (2018). Onset of foraging and lifespan of Africanized honey bees (*Apis mellifera*) infected with different levels of *Nosema ceranae* spores in neotropical Mexico. Apidologie.

[16] Antúnez K (2009). Immune suppression in the honey bee (*Apis mellifera*) following infection by *Nosema ceranae* (Microsporidia). Environ. Microbiol..

[17] Chaimanee V, Chantawannakul P, Chen Y, Evans JD, Pettis JS (2012). Differential expression of immune genes of adult honey bee (*Apis mellifera*) after inoculated by *Nosema ceranae*. J. Insect Physiol..

[18] Garrido PM (2016). Sublethal effects of acaricides and *Nosema ceranae* infection on immune related gene expression in honeybees. Vet. Res..

[19] Li W, Chen Y, Cook SC (2018). Chronic *Nosema ceranae* infection inflicts comprehensive and persistent immunosuppression and accelerated lipid loss in host *Apis mellifera* honey bees. Int. J. Parasitol..

[20] Tesovnik T (2020). Exposure of honey bee larvae to thiamethoxam and its interaction with *Nosema ceranae* infection in adult honey bees. Environ. Pollut..

[21] Li W (2017). Spore load and immune response of honey bees naturally infected by *Nosema ceranae*. Parasitol. Res..

[22] Sinpoo C, Paxton RJ, Disayathanoowat T, Krongdang S, Chantawannakul P (2018). Impact of *Nosema ceranae* and *Nosema apis* on individual worker bees of the two host species (*Apis cerana* and *Apis mellifera*) and regulation of host immune response. J. Insect Physiol..

[23] Plischuk S (2009). South American native bumblebees (Hymenoptera: Apidae) infected by *Nosema ceranae* (Microsporidia), an emerging pathogen of honeybees (*Apis mellifera*). Environ. Microbiol. Rep..

[24] Li J (2012). Diversity of Nosema associated with bumblebees (*Bombus* spp.) from China. Int. J. Parasitol..

[25] Graystock P, Yates K, Darvill B, Goulson D, Hughes WO (2013). Emerging dangers: Deadly effects of an emergent parasite in a new pollinator host. J. Invertebr. Pathol..

[26] Arbulo N (2015). High prevalence and infection levels of *Nosema ceranae* in bumblebees *Bombus atratus* and *Bombus bellicosus* from Uruguay. J. Invertebr. Pathol..

[27] Sinpoo C, Disayathanoowat T, Williams PH, Chantawannakul P (2019). Prevalence of infection by the microsporidian *Nosema* spp. in native bumblebees (*Bombus* spp.) in northern Thailand. PLoS ONE.

[28] Purkiss T, Lach L (2019). Pathogen spillover from *Apis mellifera* to a stingless bee. Proc. R. Soc. B Biol. Sci..

[29] Porrini MP (2017). *Nosema ceranae* in South American native stingless bees and social wasp. Microb. Ecol..

[30] Graystock P, Goulson D, Hughes WO (2015). Parasites in bloom: Flowers aid dispersal and transmission of pollinator parasites within and between bee species. Proc. R. Soc. B Biol. Sci..

[31] Lundin O, Rundlöf M, Smith HG, Fries I, Bommarco R (2015). Neonicotinoid insecticides and their impacts on bees: A systematic review of research approaches and identification of knowledge gaps. PLoS ONE.

[32] Lämsä J, Kuusela E, Tuomi J, Juntunen S, Watts PC (2018). Low dose of neonicotinoid insecticide reduces foraging motivation of bumblebees. Proc. R. Soc. B Biol. Sci..

[33] Morfin N, Goodwin PH, Hunt GJ, Guzman-Novoa E (2019). Effects of sublethal doses of clothianidin and/or *V. destructor* on honey bee (*Apis mellifera*) self-grooming behavior and associated gene expression. Sci. Rep..

[34] Di Prisco G (2013). Neonicotinoid clothianidin adversely affects insect immunity and promotes replication of a viral pathogen in honey bees. Proc. Natl. Acad. Sci..

[35] Tarek H, Hamiduzzaman MM, Morfin N, Guzman-Novoa E (2018). Sub-lethal doses of neonicotinoid and carbamate insecticides reduce the lifespan and alter the expression of immune health and detoxification related genes of honey bees (*Apis mellifera*). Genet. Mol. Res..

[36] Morfin N, Goodwin PH, Guzman-Novoa E (2020). Interaction of field realistic doses of clothianidin and *Varroa destructor* parasitism on adult honey bee (*Apis mellifera* L.) health and neural gene expression, and antagonistic effects on differentially expressed genes. PLoS ONE.

[37] Alaux C (2010). Interactions between *Nosema* microspores and a neonicotinoid weaken honeybees (*Apis mellifera*). Environ. Microbiol..

[38] Vidau C (2011). Exposure to sublethal doses of fipronil and thiacloprid highly increases mortality of honeybees previously infected by *Nosema ceranae*. PLoS ONE.

[39] Retschnig G, Neumann P, Williams GR (2014). Thiacloprid-*Nosema ceranae* interactions in honey bees: Host survivorship but not parasite reproduction is dependent on pesticide dose. J. Invertebr. Pathol..

[40] Doublet V, Labarussias M, de Miranda JR, Moritz RFA, Paxton RJ (2015). Bees under stress: sublethal doses of a neonicotinoid pesticide and pathogens interact to elevate honey bee mortality across the life cycle: Pesticide-pathogen interactions in honey bees. Environ. Microbiol..

[41] Gregorc A (2016). Effects of *Nosema ceranae* and thiamethoxam in *Apis mellifera*: A comparative study in Africanized and Carniolan honey bees. Chemosphere.

[42] Rosa A (2016). Consumption of the neonicotinoid thiamethoxam during the larval stage affects the survival and development of the stingless bee, *Scaptotrigona* aff. *depilis*. Apidologie.

[43] Moreira DR (2018). Toxicity and effects of the neonicotinoid thiamethoxam on *Scaptotrigona bipunctata* lepeletier, 1836 (Hymenoptera: Apidae). Environ. Toxicol..

[44] Ayala R, Gonzalez VH, Engel MS, Vit P, Pedro SR, Roubik D (2013). Mexican stingless bees (Hymenoptera: Apidae): Diversity, distribution, and indigenous knowledge. Pot-Honey.

[45] Contreras-Escareño F, Echazarreta CM, Guzman-Novoa E, Macías-Macías JO (2019). Traditional knowledge and potential use of stingless bees (Hymenoptera: Meliponinae) in the manantlan sierra, Jalisco, Mexico. Sociobiology.

[46] Macías-Macías JO, Tapia-Gonzalez JM, Contreras-Escareño F (2016). Foraging behavior, environmental parameters and nests development of *Melipona colimana* Ayala (Hymenoptera: Meliponini) in temperate climate of Jalisco, Mexico. Braz. J. Biol..

[47] Tapia-González JM (2019). Evidence of presence and replication of honey bee viruses among wild bee pollinators in subtropical environments. J. Invertebr. Pathol..

[48] Macías-Macías JO, Quezada-Euan JJG, Tapia-Gonzalez JM, Contreras-Escareño F (2014). Nesting sites, nest density and spatial distribution of *Melipona colimana* Ayala (Hymenoptera: Apidae: Meliponini) in two highland zones of western, Mexico. Sociobiology.

[49] Kessler SC (2015). Bees prefer foods containing neonicotinoid pesticides. Nature.

[50] Arce AN (2018). Foraging bumblebees acquire a preference for neonicotinoid-treated food with prolonged exposure. Proc. R. Soc. B Biol. Sci..

[51] Guimarães-Cestaro L (2020). Occurrence of virus, microsporidia, and pesticide residues in three species of stingless bees (Apidae: Meliponini) in the field. Sci. Nat..

[52] Furlong MJ, Groden E (2001). Evaluation of synergistic interactions between the Colorado potato beetle (Coleoptera: Chrysomelidae) pathogen *Beauveria bassiana* and the insecticides imidacloprid and cyromazine. J. Econ. Entomol..

[53] Alizadeh, A., Samih, M. A. & Izadi, H. Compatibility of *Verticillium lecani* (Zimm.) with several pesticides. *Commun. Agric. Appl. Biol. Sci*. **72**, 1011–1015 (2007). 18396843

[54] Pettis JS, vanEngelsdorp D, Johnson J, Dively G (2012). Pesticide exposure in honey bees results in increased levels of the gut pathogen *Nosema*. Naturwissenschaften.

[55] Williams GR, Shutler D, Burgher-MacLellan KL, Rogers RE (2014). Infra-population and community dynamics of the parasites *Nosema apis* and *Nosema ceranae*, and consequences for honey bee (*Apis mellifera*) hosts. PLoS ONE.

[56] Emsen B (2016). Higher prevalence and levels of *Nosema ceranae* than *Nosema apis* infections in Canadian honey bee colonies. Parasitol. Res..

[57] Retschnig G (2015). Effects, but no interactions, of ubiquitous pesticide and parasite stressors on honey bee (*Apis mellifera*) lifespan and behaviour in a colony environment. Environ. Microbiol..

[58] Texier C, Vidau C, Viguès B, El Alaoui H, Delbac F (2010). Microsporidia: A model for minimal parasite–host interactions. Curr. Opin. Microbiol..

[59] Hoch G, Solter LF, Schopf A (2004). Hemolymph melanization and alterations in hemocyte numbers in *Lymantria dispar* larvae following infections with different entomopathogenic microsporidia. Entomol. Exp. Appl..

[60] Gilliam M, Shimanuki H (1967). In vitro phagocytosis of *Nosema apis* spores by honey-bee hemocytes. J. Invertebr. Pathol..

[61] Ravaiano SV, Barbosa WF, Tomé HVV, de Campos LAO, Martins GF (2018). Acute and oral exposure to imidacloprid does not affect the number of circulating hemocytes in the stingless bee *Melipona quadrifasciata* post immune challenge. Pestic. Biochem. Physiol..

[62] Brandt A, Gorenflo A, Siede R, Meixner M, Büchler R (2016). The neonicotinoids thiacloprid, imidacloprid, and clothianidin affect the immunocompetence of honey bees (*Apis mellifera* L.). J. Insect Physiol..

[63] Gary NE, Lorenzen K (1990). Vacuum devices for capturing and partitioning commingled subpopulations of honey bees (Hymenoptera: Apidae). Ann. Entomol. Soc. Am..

[64] Cantwell GE (1970). Standard methods for counting *Nosema* spores. Am. Bee J..

[65] McGowan J (2016). Viability and infectivity of fresh and cryopreserved *Nosema ceranae* spores. J. Microbiol. Methods.

[66] Hamiduzzaman M, Guzman-Novoa E, Goodwin PH (2010). A multiplex PCR assay to diagnose and quantify *Nosema* infections in honey bees (*Apis mellifera*). J. Invertebr. Pathol..

[67] Macías-Macías JO, Quezada-Euán JJG, González JMT (2011). Effect of lodging type on the internal temperature and humidity of colonies of *Melipona colimana* (Hymenoptera: Meliponini) from a Mexican temperate zone. J. Apic. Res..

[68] Morfin N, Goodwin PH, Correa-Benitez A, Guzman-Novoa E (2019). Sublethal exposure to clothianidin during the larval stage causes long-term impairment of hygienic and foraging behaviours of honey bees. Apidologie.

[69] Laurino D, Manino A, Patetta A, Porporato M (2013). Toxicity of neonicotinoid insecticides on different honey bee genotypes. Bull. Insectol..

[70] Koleoglu G, Goodwin PH, Reyes-Quintana M, Hamiduzzaman MMd, Guzman-Novoa E (2018). *Varroa destructor* parasitism reduces hemocyte concentrations and prophenol oxidase gene expression in bees from two populations. Parasitol. Res..

